# Case report: Fatal outcome of pyridoxine-dependent epilepsy presenting as respiratory distress followed by a circulatory collapse

**DOI:** 10.3389/fped.2022.940103

**Published:** 2022-07-28

**Authors:** Giulia Aquilano, Agnes Linnér, Sofia Ygberg, Tommy Stödberg, Ewa Henckel

**Affiliations:** ^1^Department of Neonatology, Karolinska University Hospital, Stockholm, Sweden; ^2^Department of Clinical Science, Intervention and Technology, Karolinska Institutet, Stockholm, Sweden; ^3^Department of Child Neurology, Karolinska University Hospital, Stockholm, Sweden; ^4^Department of Women's and Children's Health, Karolinska Institutet, Stockholm, Sweden; ^5^Center for Inherited Metabolic Diseases (CMMS), Karolinska University Hospital, Stockholm, Sweden

**Keywords:** pyridoxine-dependent epilepsy, PDE, neonatal encephalopathy, lactic acidosis, neonatal respiratory distress

## Abstract

Pyridoxine-dependent epilepsy is a rare autosomal recessive disease usually associated with neonatal seizures that do not respond to common antiseizure medications but are controlled by pyridoxine administration. Because the symptoms can mimic common neonatal disorders, the diagnosis can be initially missed or delayed. We report a fatal case of a boy who was initially diagnosed with respiratory distress, birth asphyxia, and persistent pulmonary hypertension and whose condition rapidly deteriorated during the first day of life.

## Introduction

Pyridoxine-dependent epilepsy (PDE; OMIM 266100) is a rare disease firstly described in 1954 by Hunt et al. (1). The classical clinical onset is with seizures within the first month of life, often within hours after birth (2) that evolve into encephalopathy and status epilepticus despite adequate treatment with antiseizure medications (3). However, the clinical spectrum is broad, and the time of clinical onset is variable. Onset can already occur during pregnancy with abnormal fetal movements or cerebral anomalies, but cases of seizures and autistic features developing after the first year of life (late-onset) have also been reported (4).

The neurological symptoms might not be the main clinical signs and PDE can even present with a wide range of other clinical features, including respiratory distress, abdominal distension, bilious vomiting, hepatomegaly, hypothermia, hypoglycemia, shock, and acidosis (4).

Thus, PDE can be easily confused with other common neonatal conditions, such as hypoxic-ischemic encephalopathy or sepsis due to the age of onset and multisystem signs (3).

## Case report

A boy was born at 41 weeks and 3 days of gestational age by vaginal delivery after induction of labor because of pathological cardiotocography during the latent phase. This was the first child to non-consanguineous parents, after uncomplicated pregnancy with normal routine ultrasound at 12 and 20 weeks of gestation. The baby had meconium-stained amniotic fluid, with a birth weight of 4,300 grams, Apgar score 9 at 1 min and 10 at 5 min and normal umbilical cord blood gas analysis (artery: pH 7.21, pCO_2_ 7.20 kPa (54 mmHg), pO_2_ 2 (15 mmHg) BE −6 mmol/L; vein: pH 7.21, pCO_2_ 7.36 kPa (55.2 mmHg), pO_2_ 1.8 kPa (13.5 mmHg), BE −5 mmol/L). At 2 h of life, the infant was noted to be pale and in poor general condition with respiratory distress and oxygen desaturations down to 75%. Saturation improved rapidly after administration of nasal continuous positive airway pressure (nCPAP) of 4 cmH_2_0 and stabilized on saturation levels above 95% with oxygen up to 50%. A peripheral venous blood gas sample showed acidosis with a pH of 6.85, pCO_2_ 9,1 kPa (68 mmHg), BE −21 mmol/L, and lactic acid of 18 mmol/L. Clinical findings included increased work of breathing with intercostal retractions that were improved after application of nCPAP, pale skin color, normal body temperature, normal muscle tone, and soft abdominal distension. The child received a nasogastric tube to remove air from the stomach, and central umbilical lines were inserted. Antibiotics were started, and a blood gas taken from the umbilical artery confirmed the metabolic acidosis. Despite a slight improvement in arterial blood gases, the baby showed desaturation when disturbed, needed more oxygen, and partial oxygen pressure (PaO_2_) was kept around 10 kPa (75 mmHg). Oxygen levels could be reduced after air was removed continuously from the stomach and 10 ml/kg of saline bolus was given intravenously. The baby was immediately transferred to a tertiary neonatal intensive care unit (NICU).

Upon arrival, the baby was on nCPAP support with 70% oxygen, pale, grunting, and tachypneic with intercostal retractions. Physical examination revealed no heart murmur, normal femoral pulses, irritability, and increased muscle tone. No seizures were seen. The blood pressure was normal (45 mmHg). A chest x-ray ruled out a pneumothorax and other major anomalies. A new blood gas sample showed pH 7,17, pCO_2_ 6,5 kPa (48.8 mmHg), BE −12 mmol/L, and lactic acid 9,8 mmol/L. On the clinical suspicion of persistent pulmonary hypertension of the newborn (PPHN), the infant was started on clonidine 0,35 microg/kg/h for mild sedation. Because of poor response, the infusion was increased to 0,5 micro/kg/h and a 50 microg/kg bolus midazolam was administered. Right after the midazolam administration, the baby became less irritable, and the oxygen requirement could be decreased to 50% but at the same time, the blood pressure began to lower. Abnormal myoclonic movements of the arms and legs were also noted, and an EEG monitoring was requested to rule out epileptic seizures. While placing the scalp EEG electrodes, the blood pressure dropped significantly, the baby became cyanotic and unresponsive to increased oxygen supplementation. Despite intubation, ventilation, and full attempts of resuscitation (administration of a saline bolus, atropine, adrenalin, dopamine, dobutamine, and heart compressions), the baby died at about 15 h of age.

A postmortem examination revealed no anatomical anomalies and ruled out signs of air emboli. The brain was normal without signs of asphyxia. Bacterial and viral infections were not detected. The unexpected death of this child led to the suspicion of an underlying disease. Saved plasma from when the baby was alive was used to screen for metabolic diseases. Plasma acylcarnitine and amino acids analysis were unrevealing, but the concentration of plasma pipecolic acid was increased (10,6 micro mol/L, normal values <2,4). Whole-genome sequencing (WGS) was performed on DNA from cutaneous fibroblasts. Analysis of 943 known disease genes related to metabolic disorders and other monogenic disorders with similar symptomatology, revealed a homozygous known pathogenic missense variant, c.1279G>C, p. Glu427Gln, in the *ALDH7A1* gene. Sanger sequencing confirmed the finding and both parents were heterozygous carriers in blood. No history of the disease was found in the parental pedigrees. Pre-implantation genetic testing is available for this disease and genetic counseling was offered.

## Discussion

We report a fatal PDE case of a boy who was initially diagnosed with respiratory distress, birth asphyxia, and possible persistent pulmonary hypertension and whose condition rapidly deteriorated during the first day of life. The suspicion of clinical seizures arose late in the course and circulatory collapse and death ensued before an EEG was acquired. Irritability and increased muscle tone were present early, but eventual severe encephalopathy or other signs of neurological or metabolic disease were obscured by the respiratory distress.

Pyridoxine-dependent epilepsy (PDE) is a rare autosomal recessive disorder caused by biallelic mutations in the gene *ALDH7A1* coding for aldehyde dehydrogenase 7A1, also known as antiquity (ATQ). ATQ is involved in lysine catabolism and its dysfunction results in the accumulation of neurotoxic metabolic intermediates of the lysine catabolism pathway, including α-aminoadipic semialdehyde α-(AASA), piperideine-6-carboxylate (P6C), and pipecolic acid (PA). These metabolites determine a chemical inactivation of pyridoxal phosphate (PLP) resulting in a functional lack of PLP (5).

The most common findings are neurological symptoms, such as hypotonia, hypertonia, irritability, abnormal cry, exaggerated startle response, dystonic movements, and typically seizures (focal, generalized, infantile spasms, and status epilepticus) (3). EEG findings are non-specific, and abnormalities range from a mildly slow background activity to generalized, multifocal epileptiform activity, hypsarrhythmia, and burst suppression pattern (4); the latter being seen in the most severe neonatal onset. Brain anomalies can also be associated (ventriculomegaly, cortical atrophy, white matter anomalies, and incomplete/delayed myelinization).

The diagnosis of PDE is suspected when seizures are responsive to pyridoxine administration, and it is confirmed by genetic analysis showing homozygous or compound heterozygous mutations in the antiquitin gene *ALDH7A1*. The measurements of multiple lysine metabolites in urine, plasma, and cerebrospinal fluid (CSF) provide valuable information and can be used as early diagnostic markers of PDE (6). Some PDE cases do not satisfactorily respond to pyridoxine but to folinic acid, and it has been suggested that combination therapy with pyridoxine and folinic acid, and also with lysine reduction therapy (lysine restricted diet and/or L-arginine supplementation), could improve cognitive outcome (7–10).

Despite early diagnosis and effective control of seizures, significant neurodevelopmental impairment and intellectual disability have been identified in more than 70% of patients (11). In untreated patients, fatal outcome within 2 days and 16 months of age (12), but also as early as within 1 hour from birth (13), has been described.

Low Apgar scores, respiratory distress, abdominal distension, bilious vomiting, hepatomegaly, hypothermia, and shock have been described in patients with PDE (4). However, these signs are not specific. In addition, abnormal cord blood gases with acidosis and lactic acidosis, and other abnormalities of blood chemistry like hypoglycemia and profound electrolyte disturbances, have been described in PDE (4, 11, 14). As most of these disturbances have improved upon pyridoxine treatment, a direct relation to ATQ deficiency is plausible (7).

Moreover, seizures may not be the main clinical feature or, in cases, may not be present at all, making the diagnosis even more complex. Affected neonates may experience periods of encephalopathy (irritability, crying, fluctuating tone, poor feeding, and coma), sometimes associated with a burst suppression pattern on the EEG, that precede the onset of clinical seizures (15).

Nabbout and colleagues described the case of 4 infants where the seizures were not the first clinical sign. Irritability was a common sign but one patient was diagnosed with intestinal ileus and the onset of seizures was only after 3 days, while 2 patients had signs of fetal distress (meconium-stained fluid) before showing clinical signs of seizures (13). In a study by Mills and colleagues, 33% of patients (6/18) had signs of respiratory distress (4).

Sometimes, a partial or temporary response to conventional antiseizure medications can be seen in PDE, which might delay the diagnosis. In addition, the response to pyridoxine is not always prompt and at least a 72 h of trial is recommended while waiting for the results of the diagnostic workup (4).

Clinical protocols for neonatal seizures include treatment with pyridoxine intravenously if seizures are resistant to first- or second-line antiseizure medications. In our case seizures were first suspected at 12 h of age. The respiratory distress was the main clinical sign, and together with a deep metabolic acidosis and irritability, was suggestive of postnatal asphyxia and/or abnormal transition as with pulmonary hypertension. The clinical course of our patient was rapidly fatal, and the boy died within 15 h from birth before seizures could be confirmed. PDE was not suspected and, therefore, alternative therapies were not tested. Postmortem workup showed an increased plasma concentration of pipecolic acid, and WGS revealed a homozygous pathogenic variant in the *ALDH7A1* gene, thus, confirming the PDE diagnosis. The p. Glu427Gln variant is the most commonly known pathogenic *ALDH7A1* variant accounting for more than 30% of all pathological variants, and functional studies have shown complete loss of enzyme activity (16, 17).

The p.Glu427Gln variant has been referred to as a mutational hotspot of the disease (18) and the region of ALDH7A1, where this variant is located, is considered to be essential to protein folding and stability (19). In addition, other variants characterized by an amino acid change in the same position (pGlu427Gly) have been associated with the disease suggesting that a change in this position may not be tolerated (18).

No genotype-phenotype correlations have been identified, and homozygous p. Glu427Gln variants have been observed in both neonatal and late onset PDE (15, 18).

The rate of intrauterine demise and neonatal mortality for PDE has not been established but recent studies suggest that the incidence could be 6 to 12 times higher than that recognized on clinical grounds (16). PDE could, therefore, be an encephalopathy potentially misdiagnosed. It has been suggested that an earlier onset of clinical seizures corresponds to a worse prognosis for cognitive function, but also that a long delay in diagnosis and initiation of effective pyridoxine treatment correlates with increased impairments (2, 20). WES and genome sequencing (WGS) are available for early etiological diagnosis of epilepsy (21). At our hospital, acute genetic diagnostics with WGS results within 72 h is available for intensive care cases of metabolic disease and epileptic encephalopathies like PDE (22). However, this would not have helped us in this fatal case with acute onset and rapid course.

In addition to PDE, two more vitamin B6-related monogenic disorders with similar presentations have been described. Pyridoxamine 5'-phosphate oxidase deficiency (PNPOD; OMIM 610090) is caused by biallelic mutations in the pyridoxamine 5-prime-phosphate oxidase gene (*PNPO*) (23). PNPO converts both pyridoxine phosphate and pyridoxamine phosphate to pyridoxal phosphate (PLP), which is the active form of vitamin B6 and is only available for enteral administration. PNPOD responds best to PLP, and some centers advocate PLP as the first-line treatment in suspected PDE to cover also PNPOD, while others recommend PLP as the second line when pyridoxine response is insufficient. Recently, another early-onset vitamin B6-dependent epilepsy was described (OMIM 617290), caused by biallelic mutations in the gene *PLPBP* coding for a pyridoxal phosphate–binding protein that regulates the intracellular homeostasis of PLP (24). Just as in PNPOD, there is a lack of active PLP, patients respond primarily to PLP but sometimes to pyridoxine, and the biomarkers for PDE (AASA and pipecolic acid) are not elevated.

Our case report highlights that PDE may present with early symptoms, such as encephalopathy, abdominal distension, and respiratory distress, without clinical seizures. We hypothesize that the symptoms the boy has developed were related to PDE but can only speculate if early pyridoxine administration would have changed the rapid course and fatal outcome.

We suggest that in cases like this, with multi-systemic signs of uncertain cause and insufficient response to conventional treatment, an underlying etiology should be suspected, and work-up for metabolic disease and neonatal epileptic encephalopathy be performed (Table 1). The thresholds for EEG and trial treatment with pyridoxine and other cofactors, for example, PLP and folinic acid, should be low. Rapid genetic workup is available, covering all known disease genes.

**Table 1:** Suggested acute treatment and diagnostic sampling upon suspicion of pyridoxine-dependent epilepsy (PDE) with or without seizures in a neonate.

1	Sample blood (3 ml) and urine (2–5 ml) for analysis of acylcarnitines, aminoacids, and organic acids and, in addition, specifically request analyses of AASA in urine and/or plasma or plasma pipecolic acid
2	Pyridoxine 100 mg iv can be given every 5 min until response, maximum 500 mg. Close surveillance with EEG, ECG, blood pressure, and respiration. Responders can react with respiratory and circulatory depression. Some centers do not give more than 100 mg.
3	Response or not in step 2, always continue pyridoxine treatment 100 mg per day for at least 72 h. If no response after 48 h, consider changing treatment to enteral pyridoxal phosphate (PLP) if available and consider adding folinic acid 5 mg/kg.
4	In the severely ill child, consider early rapid genetic work-up aiming at metabolic disorders and epileptic encephalopathies including the three known vitamin B6 disorders.
5	If PDE diagnosis is confirmed, consider starting lysine restricted diet and/or L-arginine supplementation

With this report, we hope to increase the awareness of these and similar potentially life-threatening diseases where early intervention may change the outcome.

## Data availability statement

The original contributions presented in the study are included in the article/supplementary material, further inquiries can be directed to the corresponding author/s.

## Ethics statement

Ethical review and approval was not required for the study on human participants in accordance with the local legislation and institutional requirements. Written informed consent to participate in this study was provided by the participants' legal guardian/next of kin.

## Author contributions

GA wrote the case report under the supervision of EH. AL, SY, and TS contributed to the manuscript writing. All authors approved of the final manuscript.

## Conflict of interest

The authors declare that the research was conducted in the absence of any commercial or financial relationships that could be construed as a potential conflict of interest.

## Publisher's note

All claims expressed in this article are solely those of the authors and do not necessarily represent those of their affiliated organizations, or those of the publisher, the editors and the reviewers. Any product that may be evaluated in this article, or claim that may be made by its manufacturer, is not guaranteed or endorsed by the publisher.
